# Microaggressions towards people affected by mental health problems: a scoping review

**DOI:** 10.1017/S2045796019000763

**Published:** 2019-12-16

**Authors:** S. Barber, P. C. Gronholm, S. Ahuja, N. Rüsch, G. Thornicroft

**Affiliations:** 1 Centre for Global Mental Health, and Centre for Implementation Science, Institute of Psychiatry, Psychology and Neuroscience, King's College London, London, UK; 2 Department of Psychiatry II, University of Ulm and BKH Günzburg, Ulm, Germany

**Keywords:** Community mental health, discrimination, mental health, mental illness stigma

## Abstract

**Aims:**

This review aims to understand the scope of the literature regarding mental health-related microaggressions towards people affected by mental health problems.

**Methods:**

A scoping review was conducted to explore this question. Four electronic health-oriented databases were searched alongside Google Scholar. As per scoping review principles, the inclusion criteria were developed iteratively. The results of included studies were synthesised using a basic narrative synthesis approach, utilising principles of thematic analysis and thematic synthesis where appropriate.

**Results:**

A total of 1196 records were identified, of which 17 met inclusion criteria. Of these, 12 were peer-reviewed journal articles, three were research degree theses and two were book chapters. Six included empirical studies were qualitative, four were quantitative and two employed a mixed-methods design. Within these, five qualitative studies aimed to describe the nature of mental health microaggressions experienced by people with mental health problems. Themes identified in a thematic synthesis of these five studies included stereotypes about mental illness, invalidating peoples' experience and blaming people with mental illness for their condition. The included publications informed on the perpetration of mental health microaggressions by family, friends, health professionals and social workers. In addition, two studies created scales, which were then used in cross-sectional surveys of the general public and community members to assess characteristics, such as right-wing political views, associated with endorsement of mental health microaggressions. A consensus definition of microaggressions emerged from the included studies: microaggressions are brief, everyday slights, snubs or insults, that may be subtle or ambiguous, but communicate a negative message to a target person based on their membership of a marginalised group, in this case, people affected by mental illness.

**Conclusions:**

The study of mental health microaggressions is an emerging, heterogeneous field, embedded in the wider stigma and discrimination literature. It has been influenced by earlier work on racial microaggressions. Both can be ambiguous and contradictory, which creates difficulty defining the boundaries of the concept, but also underpins the key theoretical basis for the negative impact of microaggressions. Mental illness is a more concealable potential type of identity, so it follows that the reported perpetrators of microaggressions are largely friends, family and professionals. This has implications for intervening to reduce the impact of microaggressions. There are several challenges facing research in this area, and further work is needed to understand the impact of mental health microaggressions on people affected by mental health problems.

## Introduction

The term ‘microaggression’ was coined in 1970 to describe subtle dismissals and insults towards Black Americans (Pierce, 1970). The word remained largely in obscurity until the late 2000s, when a paper exploring ‘subtle and contemporary’ forms of racism in the context of therapy gained widespread public attention (Sue *et al*., 2007). Based on the analysis of personal narratives and the social and counselling psychology literature, Sue *et al.* defined microaggressions as ‘brief and commonplace daily verbal, behavioural or environmental indignities, whether intentional or unintentional, that communicate hostile, derogatory, or negative racial slights and insults towards people of color’ (Sue *et al*., 2007, p. 271). They describe three distinct forms of microaggressions: microassault, microinsult and microinvalidation. A microassault is the most overt form, for example, intentionally calling a person of colour a derogatory term. Microinsults are more subtle and convey rudeness or insensitivity, for example, clutching one's purse more tightly when in the presence of a person of colour. Finally, microinvalidations negate or nullify the feelings or experiences of a person, by saying, for example, ‘Don't be so oversensitive’ (Sue *et al*., 2007, p. 275).

The concept of subtle, contemporary forms of racism has captured the attention of wide audiences, not least because these researchers argue that microaggressions have a powerful, negative cumulative effect on the mental health of people of colour (Sue *et al*., 2007; Sue, 2010). The theoretical basis of this assertion is grounded in ‘minority stress theory’ (Meyer, 1995) which states that a hostile social environment demands heightened vigilance to protect oneself from discrimination and violence. It is also argued that the subtle nature of microaggressions creates a ‘catch-22’ (or ‘no win’) situation, in which an individual is left questioning the validity of his or her experience (‘Did what I think happened, really happen?’). This concept has been described as attributional ambiguity in the wider stigma literature and is argued to pose a particular threat to self-esteem and lead to suspicion and mistrust (Crocker *et al*., 1998).

Numerous studies have reported a significant correlation between reported experiences of microaggressions and self-reported negative mental health outcomes (e.g. Nadal *et al*., 2014; Gattis and Larson, 2017; Reid, 2017). Whilst most studies have been cross-sectional, a study using a longitudinal design has shown that microaggressions in the form of ‘underestimation of personal ability’ significantly predicted self-rated depression symptoms 1 year later – providing some indication of potential causation (Torres *et al*., 2010).

Research on microaggression is not without its critics. Some argue that a microaggression is an open concept with ‘fuzzy’ boundaries, full of contradictions and ambiguities – for example, both ignoring and attending to minority students in classrooms have been listed as microaggressions (Lilienfeld, 2017). It is apparent that microaggressions are ambiguous and ‘lie in the eye of the beholder’, and so, variations in responses by targets of microaggressions may be a function of an individual's personality dispositions, a key confounder when asserting a causal association between microaggressions and adverse mental health outcomes (Lilienfeld, 2017). Indeed, once accounting for scores of ‘perceived stress’ (feeling life is unpredictable, uncontrollable and overloaded), Torres *et al.* found that the predictive effect of microaggressions on depression symptoms was non-significant (Torres *et al*., 2010).

Nevertheless, the use of the term ‘microaggression’ is expanding. It is now used to describe snubs, slights and insults towards members of other marginalised social groups including women, sexual minorities and people with disabilities (Sue, 2010). There is a growing literature on the experience of people affected by mental health problems, arguably a marginalised social group in its own right. This emerging field can be seen to fit into the wider literature on stigma towards mental illness – a broad concept that encompasses ignorance, prejudice and discrimination (Thornicroft, 2006).

The purpose of this scoping review was to answer the following research question: ‘What is the scope of the literature regarding mental health-related microaggressions towards people affected by mental health problems?’. We aimed to characterise the literature in this area (e.g. who is conducting this research, and from what discipline?), elucidate the purpose of research on this topic for this population (e.g. to describe the experiences of people with mental health problems or to measure the effect) and describe and synthesise the current evidence base. In doing so, we planned to clarify the definition of the term microaggression as it is used in relation to people with mental health problems. In addition, we aimed to identify the problematic areas of this research topic (e.g. varying definitions, the distinction between a microaggression and other forms of subtle discrimination) and propose the next steps for researchers in this field.

## Methods

A scoping review approach was used to answer the research question. Like a systematic review, a scoping review is informed by an *a priori* protocol, involves systematic and exhaustive searching, must be transparent and reproducible, includes steps to reduce error and increase reliability, and presents data in a structured way (Munn *et al*., 2018). However, in contrast to the systematic review method which is guided by a highly focussed research question, the scoping review method is guided by a requirement to identify all relevant literature, and as such it allows for procedural flexibility during the conduct of the review to achieve this aim (Arksey and O'Malley, 2005). A scoping review methodology was therefore considered highly appropriate for our research aim to characterise and synthesise the current literature. We followed the procedure outlined by Arksey and O'Malley (2005) and further developed by Levac *et al*. (2010).

This review complies with the preferred reporting items for systematic reviews and meta-analyses (PRISMA) statement (Moher *et al*., 2009). It follows an *a priori* developed review protocol, registered at the Open Science Framework platform (Barber *et al*., 2019).

### Identifying the review question and aims

The research question was formulated through consideration of the concept and target population, using aspects of the SPIDER question format tool (i.e. examining Sample, Phenomenon of Interest, Design, Evaluation, and Research type) (Cooke *et al*., 2012). For this review, we specified only the ‘sample’ and ‘phenomenon of interest’ aspects, to fulfil the aim of characterising the literature in this area.

In line with the iterative, flexible nature of the scoping review process (Levac *et al*., 2010), the ‘sample’ was revised during the study selection process. Initially defined as ‘people with mental health problems’, this was refined to ‘people whose defining characteristic, in the study, is being affected by a mental health problem or disability’.

The ‘phenomenon of interest’ was mental health-related microaggressions specifically. This was specified as microaggressions attributed to a mental health problem or mental health-related disability. We did not include papers which addressed racial or other microaggressions directed towards people with mental health problems. Unless otherwise stated, the term microaggression in this manuscript refers to this narrow definition.

### Identifying relevant studies

The search strategy was designed to identify formally published, peer-reviewed articles and selected grey literature, including research degree theses and book chapters but not ‘grey data’ (e.g. websites, tweets and blog posts). Four electronic health-oriented databases were searched in July 2019: Medline, EMBASE, PsycINFO and WorldCat Dissertation database. We also searched Google Scholar, identified as a powerful addition to traditional search methods (Haddaway *et al*., 2015). The first 300 results of a search on ‘Incognito mode’ (used to improve replicability) were screened.

The search strategies consisted of keywords and subject headings related to ‘mental health’ and ‘microaggressions’. For Google Scholar the following search strategy was used: ‘(Mental* or Psych*) AND microaggress*’. Appendix 1 gives full search strategies for each database.

We supplemented the database and search engine searches by conducting backwards and forwards citation checking and contacting authors and experts in the field.

### Study selection

Study selection followed a two-step approach: (1) title, abstract, key word screening, (2) full-text screening. In both stages, SB and SA independently screened a proportion of results (25 and 40%, respectively), with PCG acting as an arbitrator. Interrator reliability was calculated and exceeded 80%. SB screened the remainder of the results. In line with scoping review principles, inclusion criteria were developed iteratively through discussion between authors.

### Data extraction and management

Data from the included full texts were extracted onto a ‘Characteristics of Included Studies’ table, including country, department/institution (lead author), publication type, study design, population of interest (sample size) and purpose (e.g. to describe the experience of microaggressions, or to validate a scale).

### Qualitative synthesis and analysis

The results of included studies were synthesised using a basic narrative synthesis approach, applying principles of thematic analysis (Braun and Clarke, 2006) and thematic synthesis (Thomas and Harden, 2008) where appropriate, given the available data and how it could best be examined to answer the review questions.

### Quality of included studies assessment

We used the Mixed Methods Appraisal Tool (MMAT) (Hong *et al*., 2018) to assess the quality of included empirical studies. This tool is designed for quality assessment in systematic reviews that include quantitative, qualitative and mixed-method studies. Articles were assigned one point for each criterion that was fulfilled, and half a point for each partially met the criterion. These points were summed to produce an index based on the proportion of total criteria met. No studies were excluded from the synthesis due to low-quality score.

## Results

**Fig. 1.**
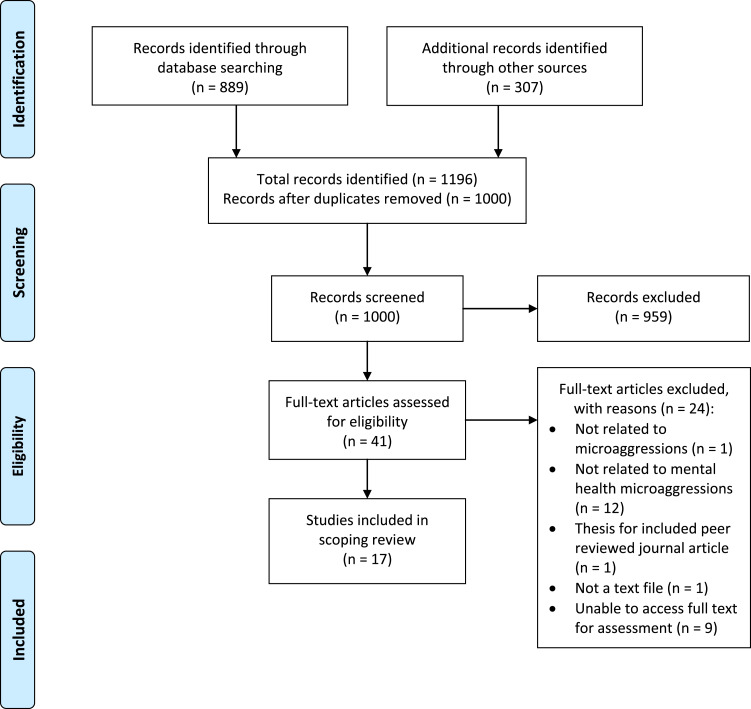
PRISMA flow diagram. Adapted from Moher *et al*. (2009).

In total, 1196 records were identified: 889 records from database searching, and 307 through other sources (300 from Google Scholar, five through communication with authors, two through consultation with experts). After duplicates were removed, 1000 records remained for title, abstract and key word screening. In total, 959 records were excluded at this stage, leaving 41 records for full-text assessment for eligibility, of which 17 met inclusion criteria for this review. This study selection procedure is illustrated in the PRISMA flow diagram (see Fig. 1).

### Characterising the literature

The characteristics of included studies are summarised in Table 1.

**Table 1.** Characteristics of included studies

Study	Country	Department/institution (of lead author)	Publication type	Study design	Population of interest (sample size)	Purpose	MMAT Index score
Gonzales *et al*. (2015a)	USA	Department of Psychology, City University of New York	Peer-reviewed journal article	Qualitative (focus groups)	People with mental health problems (21)	To describe the nature of mental health microaggressions from the perspective of people affected by mental health problems	100%
Peters *et al*. (2016)	USA	Department of Psychology, University of South Dakota	Peer-reviewed journal article	Qualitative (focus groups)	People with mental health problems (18)	To describe the nature of mental health microaggressions from the perspective of people affected by mental health problems	100%
Holley *et al*. (2016a)	USA	School of Social Work Arizona State University	Peer-reviewed journal article	Qualitative (1:1 interview)	People with mental health problem (or their family) who also identify as of colour or LGBTQ or both (20)	To examine the experience of mental illness discrimination in the mental health system	100%
McCue (2016)	USA	Unknown, Michigan State University	PhD thesis	Qualitative (1:1 interview)	Law students with mental health problems (11)	To examine the lived experiences of law students with mental health problems	100%
Zenga (2018)	USA	Faculty of the California School of Professional Psychology, Alliant International University San Diego	PhD thesis	Mixed methods: qualitative (focus groups) and cross-sectional survey	Mental health peer support specialists (16)	To examine how peer support specialists experience microaggressions	90%
Charles *et al*. (2017)	USA	School of Social Work, University of Indianapolis	Peer-reviewed journal article	Qualitative component (open text questions) of cross-sectional survey	Social work educators (246)	To determine whether social work educators report microaggressions towards students with mental illnesses, and if educators practice cultural humility	90%
Gonzales *et al*. (2015b)	USA	Department of Psychology, City University of New York	Peer-reviewed journal article	Validation of a scale	General public (505)	To develop and validate a scale for measuring microaggression behaviours as endorsed among the general population	Not applicable
Ezell *et al*. (2018)	USA	Department of Sociology, University of Chicago	Peer-reviewed journal article	Validation of a scale	People with mental health problems (65)	To develop and validate a scale for measuring the daily indignities of mental illness	Not applicable
DeLuca *et al*. (2018)	USA	Department of Psychology, City University of New York	Peer-reviewed journal article	Cross-sectional survey	General public (518)	To determine the relationship between political attitudes to multidimensional components of mental health stigma	80%
Gonzales *et al*. (2018)	USA	Department of Psychology, City University of New York	Peer-reviewed journal article	Cross-sectional survey	Community members (606), people with mental health problems (343)	To determine the relationship between microaggressions perceived by persons with disabilities and the self-reported attitudes and behaviours by community members	60%
Zurick (2016)	USA	Department of Psychology, Southern Illinois University	Masters thesis	Cross-sectional survey	General public (222)	To examine the role of specific prejudicial beliefs and intimacy of interpersonal contact in relation to mental health microaggressions	50%
DeLuca *et al*. (2017)	USA	Department of Psychology, City University of New York	Peer-reviewed journal article	Cross-sectional survey	General public (951)	To examine the influence of individual- and societal-level characteristics, including endorsement of microaggressions, on mental health funding decisions	70%
Harper *et al*. (2017)	USA	Department of Psychiatry, Yale University	Peer-reviewed journal article	Qualitative (1:1 interview)	People with mental health problems (8)	To examine the community integration experiences of people with severe mental illness	100%
Borg *et al*. (2009)	Norway	Department of Health Sciences, Buskerud University College	Peer-reviewed journal article	(Conceptual paper)	People with mental health problems in the community	To raise fundamental questions associated with user involvement in community mental health practices	Not applicable
Holley *et al*. (2016b)	USA	School of Social Work, Arizona State University	Peer-reviewed journal article	Mixed methods: qualitative (1:1 interview) and cross-sectional survey	People with mental health problems (or their family) who also identify as of colour or LGBTQ or both (20)	To examine the perceptions of similarities and differences between mental illness discrimination and racism and/or heterosexism	60%
Yanos (2017)	USA	Not stated	Book chapter	(Synthesis of literature and commentary)	People with mental health problems	To describe forms of stigmatising behaviour towards people with mental health problems	Not applicable
Holley and Thomas (2018)	Not stated	Not stated	Book chapter	(Synthesis of literature and commentary)	People with intersecting identities including mental health problems	To discuss effective social work practice	Not applicable

All papers and chapters were published in the last decade (16 in the last 4 years), 15 are from the USA (five from authors associated with the City University of New York) and most papers originated from departments of Psychology (*n*  =  8), followed by Social Work (*n*  =  3).

Six included empirical studies were qualitative in nature, using focus group (Gonzales *et al*., 2015*a*; Peters *et al*., 2016), one-to-one semi-structured interviews (Holley *et al*., 2016*a*; McCue, 2016; Harper *et al*., 2017) and analysis of free-text survey questions (Charles *et al*., 2017). Four were cross-sectional surveys (Zurick, 2016; DeLuca *et al*., 2017; DeLuca *et al*., 2018; Gonzales *et al*., 2018), with sample sizes from 222 to 951 participants. Four of these studies employed convenience sampling of the US public using online survey platforms. Two studies adopted a mixed-methods design (Holley *et al*., 2016*b*; Zenga, 2018). Two included publications aimed to validate a scale (Gonzales *et al*., 2015*b*; Ezell *et al*., 2018). The remaining three papers and chapters (Borg *et al*., 2009; Yanos, 2017; Holley and Thomas, 2018) were not research studies, but still met inclusion criteria and offered an insight into the scope of the literature.

### Characterising the purpose of research on this topic for this population

Five studies aimed to elucidate the nature of mental health microaggressions from the perspective of people affected by mental health problems (Gonzales *et al*., 2015*a*; Holley *et al*., 2016*a*; McCue, 2016; Peters *et al*., 2016; Zenga, 2018). In three, the participants had intersecting identities as racial and/or sexual orientation minority group members (Holley *et al*., 2016*a*), law students (McCue, 2016) and peer support specialists (Zenga, 2018). We synthesised the experiences of microaggressions described in these studies (see Table 2).

**Table 2.** A thematic synthesis of experiences described as mental health microaggressions

Theme (sub-theme)	Supporting quote (source)
Stereotypes about mental illness	
People with mental illness lack intelligence	‘you are not as smart or as capable to succeed or to be in like leadership positions’ (Peters *et al*., 2016, p. 93)
People with mental illness are incapable	‘because you're mentally ill, they think all you can do is just scrub floors’ (Gonzales *et al*., 2015a, p. 237)
People with mental illness are weak	‘You'll do things because someone tells you to… that you're weak.’ (Gonzales *et al*., 2015a, p. 237)
People with mental illness are dangerous	‘Well, he does have a mental illness… he tends to be violent’ (Gonzales *et al*., 2015a, p. 237)
People with mental illness are cold	‘You aren't in like this deep dark void where you don't even care about everyone else?’ (Peters *et al*., 2016, p. 96)
Treating people with mental illness differently	
Treating people with mental illness like children	‘non-[peer support specialist]staff spoke in an overly gushy tone, like to a child, I felt she was fake’ (Zenga 2018, p. 56)
Patronising people with mental illness	‘They often try to intercede on my behalf and do things for me’ (Holley *et al*., 2016a, p. 316)
Giving fake compliments to people with mental illness	‘They were trying to by nice but they weren't, pretty much. Saying like, “Wow, that's really brave of you”’ (Gonzales *et al*., 2015a, p. 237)
Ignoring people with mental illness	‘Ann described a psychiatrist's attitude during an appointment with her partner as: “I just want to ask the questions and you're going to answer [them] and that's it”’ (Holley *et al*., 2016a, p. 314)
Increasing distance from people with mental illness	‘… and they take a second look and start moving away from me, and sit somewhere else’ (Gonzales *et al*., 2015a, p. 238)
Devaluing people with mental illness	‘well we are viewed as professionals, but not quite professional’ (Zenga, 2018, p. 58)
Defining people with mental illness by their diagnosis	
Labelling people with mental illness	‘Gerard explained that staff “gear” PWMI to say “I am bipolar” rather than “I have bipolar disorder”’ (Holley *et al*., 2016a, p. 315)
Not treating people as complex individuals	‘We have thoughts, feelings, and emotions just like everyone else does’ (Holley *et al*., 2016a, p. 316)
Assuming behaviour is a symptom of mental illness	‘Or if I do a lot of activities or stay up late I'll have people call me up and say ‘Maybe you're manic, you stayed up really late. You've done a lot more things than you usually do’ (Gonzales *et al*., 2015a, p. 237)
Invalidating peoples experience of mental illness	
Doubting existence of mental illness	‘You have PTSD? Well you are acting normal’ (Zenga, 2018, p. 57)
Doubting severity of mental illness	‘You'll be fine. Just get over it’ (McCue, 2016, p. 85)
Minimizing experiences of people with mental illness	‘Oh I sometimes get sad, too’ (McCue, 2016, p. 85)
Comparing peoples' experiences of mental illness	‘I have another client who did this when they were depressed. So you're not actually that depressed’ (Gonzales *et al*., 2015a, p. 236)
Blaming people with mental illness for their condition	
People with mental illness bring it upon themselves	‘you did something to cause this to happen’ (Peters *et al*., 2016, p. 96)
Accusing people with mental illness of ulterior motives	
People use mental illness as an excuse	‘You're using it as an excuse not to do your work’ (Peters *et al*., 2016, p. 96)
People use mental illness to seek attention	‘… people treat you like you're just being dramatic’ (Peters *et al*., 2016, p. 96)
Shaming people with mental illness	
Shaming people who disclose their mental illness	‘That's okay that you're telling me but that's not appropriate to say in an interview’ (Gonzales *et al*., 2015a, p. 238)
Not acknowledging mental illness	‘I saw a picture of a relative and no one talked about her’ (Zenga, 2018, p. 55)
Misusing terminology	
Using mental health terms inappropriately	‘The weather is bipolar’ (McCue, 2016, p. 85)
Using mental health terms flippantly	‘“I feel like killing myself today” and you're like dude, no you don't’ (Peters *et al*., 2016, p. 99)

These five papers also provide data on the perpetrators of mental health microaggressions, which included family, friends and health professionals. For example, a participant in Gonzales *et al*. (2015*a*) stated: ‘People in my family, if I actually starting being happy they're like, “Are you sure you're okay?”’ (p. 237). Similarly, Peters *et al*. (2016) provided an example of friends misusing terminology ‘I even have friends that say it… they're just like, “But oh man they were so bipolar”’ (p. 100). Additionally, a participant in McCue (2016) described an experience where a fellow student said ‘that he has depression’ and that ‘he could go out and get that extra time but he has dignity’ (p. 85), consistent with perpetration of a microaggression by a peer (by shaming mental illness). One further paper aimed to describe mental health microaggressions from the perspective of potential perpetrators (Charles *et al*., 2017). They demonstrated that social work educators report personal reactions that reflect microaggressions.

Two papers aimed to create scales, one measuring the experience of microaggressions (Ezell *et al*., 2018) and one measuring endorsement of microaggressions by possible perpetrators (Gonzales *et al*., 2015*b*). The latter was used in three studies aiming to identify individual characteristics associated with endorsement of mental health microaggressions by the general public. Broadly, endorsement of microaggressions was positively associated with right-wing political views (DeLuca *et al*., 2018), suburban values and socio-economic disadvantage (Gonzales *et al*., 2018), authoritarianism (the attitude that people with serious mental illness cannot care for themselves and require coercion) and social restrictiveness (the belief that people with mental illness should be feared and excluded) (Zurick, 2016). Endorsement was negatively associated with benevolence (the belief that people with mental illness are innocent and naïve) (Zurick, 2016).

Two studies considered the impact of microaggressions. One qualitative study described a ‘negative outcome’ theme that included frustration, loss of self-esteem and alienation (Gonzales *et al*., 2015*a*). One quantitative study assessed the impact of endorsing microaggressions on preferences for mental health funding allocation by the general public and found a weak but significant negative correlation (DeLuca *et al*., 2017).

### Defining the term microaggression as it is used in relation to people with mental health problems

We found that the majority (11/17) of papers directly or indirectly referenced a definition of microaggressions from the work of Sue *et al.* (e.g. Sue, 2010; Sue *et al*., 2007), and eight listed the three types of microaggression described by Sue *et al*.: microassaults, microinsults and microinvalidations. Ezell *et al*. (2018) referenced Pierce *et al*. (1977) and described a ‘nexus of recurrent, daily indignities, both overt and non-overt, that are directed at historically marginalised populations… which insult, degrade or otherwise demoralize their recipients’ (p. 28). Borg *et al*. (2009) describe ‘the things you experience every day that then add up and take their toll’ (p. 290). Three papers provided no reference but emphasised the ‘everyday’ (Harper *et al*., 2017) or ‘subtle’ (DeLuca *et al*., 2017; DeLuca *et al*., 2018) nature of microaggressions. There is an apparent consensus across all included studies that microaggressions are brief, everyday slights, snubs or insults, which may be subtle or ambiguous, but communicate a negative message to a target person based on their membership of a marginalised group, in this case, people with experience of mental illness.

### Quality of included studies

The 12 empirical studies included in the review were assessed using the MMAT tool. Index scores are presented in Table 1. All of the qualitative studies (Gonzales *et al*., 2015*a*; Holley *et al*., 2016*a*; McCue, 2016; Peters *et al*., 2016; Charles *et al*., 2017; Harper *et al*., 2017) used an appropriate approach (interpretive or critical) and methods of data collection and analysis were well described and adequate. Overall, conclusions were well substantiated by data and the author's arguments were coherent. Of note, Charles *et al*. (2017) included a clear discussion of neutral and ambiguous findings. In general, there was an absence of reflexivity (with McCue (2016) a notable exception) or respondent validation. Three of the cross-sectional surveys used online convenience methods to sample US residents (Zurick, 2016; DeLuca *et al*., 2017, 2018). In one study, the recruitment system stratified based on demographic attributes resulting in gender balance and equal numbers of four age groups and regions (DeLuca *et al*., 2018). The other two were biased, for example, towards females. Zurick (2016) found that 36.9% of their sample reported they had a mental illness, a limitation to the study of general attitudes. All three of these surveys used validated measures and appropriate statistical techniques. One cross-sectional study used an unvalidated perceived microaggression scale (Gonzales *et al*., 2018). Two studies employed mixed methods (Zenga, 2018; Holley *et al*., 2016*b*), both using quantitative measures to triangulate the qualitative analyses. In both cases, there were weaknesses in the interpretation and integration of the quantitative findings, due to small sample size, reducing the quality of the study.

## Discussion

The results of this scoping review have demonstrated an emerging field that is highly heterogenous methodologically. Largely in the last five years, researchers have described mental health-related microaggressions, operationalised them into scales, examined the characteristics associated with perpetration and begun to study the impact. By synthesising the results of four good quality qualitative studies and one mixed-method study, we provide an overview of the experiences people with mental illness have described as microaggressions in the literature to date.

The influence of Sue *et al.* from the racial microaggression field is evident; the majority of studies directly cite their definition and some authors framed their results in terms of microassualts, microinsults and microinvalidations (Peters *et al*., 2016; McCue, 2016; Holley *et al*., 2016a; Zenga, 2018). There are similarities between racial and mental health microaggressions, such as the assumption of inferiority or dangerousness (Sue *et al*., 2007) and the notion of questioning the validity of the perceived microaggressions. For example, one focus group participant felt a false sense of support when she won an award (Gonzales *et al*., 2015*a*). This is an example of attributional ambiguity, arguably a threat to self-esteem by making it difficult to assess one's abilities (Crocker *et al*., 1998).

However, the mental health literature has key differences to the wider microaggression work. The ‘perpetrators’ of microaggressions towards people with mental illness are largely family and friends and health professionals, rather than strangers or acquaintances. This follows from mental illness being an arguably more concealable potential identity compared to race, but this has particular implications for intervening to reduce mental health microaggressions. In addition, certain experiences are unique to mental illness, such as symptomizing – where ‘emotions and behaviours considered “normal” for people without mental illness are assumed to be a symptom of their mental illness’ (Gonzales *et al*., 2015*a*, p. 236).

Criticism of the wider microaggression literature has focused on boundaries of the concept, the ambiguous and contradictory nature of microaggressions and role of unexplored confounders such as personality traits (Lilienfeld, 2017). These challenges and problems are evident in the mental health literature too. Firstly, in synthesising the experiences described as microaggressions by people affected by mental health problems, we found examples that do not fit with the apparent consensus definition of microaggressions as brief, everyday forms of discrimination. For example, ‘I was arrested, I spent 8 months of my life in a cage for something I did not do’ (Gonzales *et al*., 2015*a*, p. 237). If this incarceration had anything to do with stigma or discrimination, it might be more helpfully considered an example of structural discrimination within the legal system than a microaggression. Similarly, ‘shortly before going back to work, they informed her that someone with more experience was hired’ (Zenga, 2018, p. 59) is not consistent with a subtle slight, snub or insult.

Defining what is, and what is not, a microaggression will be critical to distinguishing microaggressions from other described forms of discrimination. Currently, authors appear to be at odds as to whether the microaggression concept is new and ‘scarcely… studied or discussed’ (Gonzales *et al*., 2015*a*, p. 234), or an extension or rebranding of previously described forms of covert discrimination or forms of oppression (e.g. Holley *et al*., 2016*a*). In his book chapter, Yanos (2017) explores the relationship between microaggressions and other forms of stigmatising behaviour. To distinguish a microaggression from a ‘social rejection’ experience, Yanos describes ‘the difference between situations in which others avoid initiating a relationship with someone and situations in which others actively end a relationship’ (p. 46). He places microaggressions on a spectrum of stigmatising behaviour, at ‘the lowest level of severity’ (p. 41).

Several papers discuss the changing, contradictory and ambiguous nature of microaggressions. For example, Charles *et al*. (2017) reflect that ‘the nature of language and what is not recognised as a microaggression is always in flux’ (p. 420) and that ‘reaching out’ to students who disclose their mental illness could be seen as either supportive or a microaggression. ‘Symptomizing’ also presents a ‘catch-22’ situation to friends, family members and health professionals. Sleep disturbances are well recognised to be manic prodromes (Sierra *et al*., 2007), and a psychoeducational family intervention (including early warning signs) has been shown to improve the level of disability of patients with bipolar disorder (Fiorillo *et al*., 2015). Yet, one participant in Gonzales *et al*. (2015*a*) gives the following example of a microaggression: ‘…if I do a lot of activities or stay up late I'll have people call me up and say “Maybe you're manic, you stayed up really late…”’ (p. 237).

The authors of a number of the included studies suggest strategies to reduce perpetration of microaggressions and their impact. They focus on health professionals (Borg *et al*., 2009; Peters *et al*., 2016; Charles *et al*., 2017), institutions (Holley *et al*., 2016*a*; McCue, 2016) and the general public (DeLuca *et al*., 2017; DeLuca *et al*., 2018), and consider training and awareness campaigns. However, given the evidence that family and friends often perpetrate microaggressions, it may be useful to target interventions to closer acquaintances, for example, in family therapy. Another paper included in this study highlighted resilience as a key theme in the experience of microaggressions by peer support specialists (Zenga, 2018). Having gained confidence through recovery, one participant explained ‘she has developed empathy and compassion because she believes that they are “trying to be supportive”’ (p. 62). Given the changing, ambiguous nature of microaggressions, supporting people with mental health problems to cope with these slights, snubs and insults may be critical to reducing the impact of microaggressions. This could be done, for example, by focusing on strategies that improve self-esteem.

### Limitations

There are several limitations to this scoping review. Firstly, our search strategy extended to grey literature but not grey data, which may have contained important perspectives. Secondly, we identified several studies in the screening stages that we were unable to access, despite attempts to contact the authors, which may represent a loss of valuable data.

We adopted a narrow focus for this scoping review for reasons of feasibility, including only papers that referenced mental health-related microaggressions explicitly. Widening the search to include terms such as subtle and covert discrimination might have enabled us to comment on distinguishing features (if they exist) of microaggressions in particular.

## Conclusions

This scoping review has revealed a highly heterogenous body of work relating to microaggressions towards people with mental illness. The research has been heavily influenced by the work of Sue *et al.* in the wider microaggression literature. However, it is also embedded in the broader field of discrimination towards people with mental illness, with apparent similarities to previously described forms of subtle discrimination such as social rejection.

We have identified many challenges in this field of research, not least issues with the definition and boundaries of the concept: what is, and what is not, a microaggression needs to be clear and distinct to other forms of discriminatory behaviour. Also, some authors have asserted that the experience of microaggressions has a negative impact on people with mental illness, with little empirical evidence. Further work, including a careful examination of potential confounding personality traits, is required (as has been done in the wider mental health stigma literature, see Schibalski *et al*. (2017)). Indeed, placing the experience of microaggressions at the ‘lowest level of severity’ (Yanos, 2017, p. 41) of stigmatising experiences may be premature. Daily exposure to subtle forms of discrimination may, paradoxically, have a greater cumulative negative effect than other forms of blatant, unambiguous discrimination (Crocker *et al*., 1998).

## Appendix 1

**Appendix 1:** Full search strategy

Database	Search strategy
Medline	1. (mental* adj5 (health* or illness* or condition* or disabilit* or disorder* or disease* or impair* or problem* or stress* or wellbeing or ‘well being’)).mp. [mp = title, abstract, original title, name of substance word, subject heading word, floating sub-heading word, keyword heading word, organism supplementary concept word, protocol supplementary concept word, rare disease supplementary concept word, unique identifier, synonyms] 2. (psych* adj5 (health* or illness* or condition* or disabilit* or disorder* or disease* or impair* or problem* or stress* or wellbeing or ‘well being’)).mp. [mp = title, abstract, original title, name of substance word, subject heading word, floating sub-heading word, keyword heading word, organism supplementary concept word, protocol supplementary concept word, rare disease supplementary concept word, unique identifier, synonyms] 3. exp Mental Health/ 4. exp Mental Disorders/ 5. 1 or 2 or 3 or 4 6. microaggress*.mp. [mp = title, abstract, original title, name of substance word, subject heading word, floating sub-heading word, keyword heading word, organism supplementary concept word, protocol supplementary concept word, rare disease supplementary concept word, unique identifier, synonyms] 7. 5 and 6
EMBASE	1. (mental* adj5 (health* or illness* or condition* or disabilit* or disorder* or disease* or impair* or problem* or stress* or wellbeing or ‘well being’)).mp. [mp = title, abstract, heading word, drug trade name, original title, device manufacturer, drug manufacturer, device trade name, keyword, floating subheading word, candidate term word] 2. (psych* adj5 (health* or illness* or condition* or disabilit* or disorder* or disease* or impair* or problem* or stress* or wellbeing or ‘well being’)).mp. [mp = title, abstract, heading word, drug trade name, original title, device manufacturer, drug manufacturer, device trade name, keyword, floating subheading word, candidate term word] 3. exp mental health/ 4. exp mental disease/ 5. 1 or 2 or 3 or 4 6. microaggress*.mp. [mp = title, abstract, heading word, drug trade name, original title, device manufacturer, drug manufacturer, device trade name, keyword, floating subheading word, candidate term word] 7. 5 and 6
PsycINFO	1. (mental* adj5 (health* or illness* or condition* or disabilit* or disorder* or disease* or impair* or problem* or stress* or wellbeing or ‘well being’)).mp. [mp = title, abstract, heading word, table of contents, key concepts, original title, tests & measures, mesh] 2. (psych* adj5 (health* or illness* or condition* or disabilit* or disorder* or disease* or impair* or problem* or stress* or wellbeing or ‘well being’)).mp. [mp = title, abstract, heading word, table of contents, key concepts, original title, tests & measures, mesh] 3. exp Mental Health/ 4. exp Mental Disorders/ 5. 1 or 2 or 3 or 4 6. microaggress*.mp. [mp = title, abstract, heading word, table of contents, key concepts, original title, tests & measures, mesh] 7. 5 and 6
WorldCat Dissertation database	kw: mental* and kw: adj5 and kw: health* or kw: illness* or kw: condition* or kw: disabilit* or kw: disorder* or kw: disease* or kw: impair* or kw: problem* or kw: stress* or kw: wellbeing or kw: well w being) OR (kw: psych* and kw: adj5 and kw: health* or kw: illness* or kw: condition* or kw: disabilit* or kw: disorder* or kw: disease* or kw: impair* or kw: problem* or kw: stress* or kw: wellbeing or kw: well w being) AND kw: microaggress*
